# Early Vessels Exploration of Pink Pulseless Hand in Gartland III Supracondylar Fracture Humerus in Children: Facts and Controversies

**DOI:** 10.5704/MOJ.1703.005

**Published:** 2017-03

**Authors:** TZ Tunku-Naziha, WMS Wan-Yuhana, D Hadizie, Muhammad-Paiman, S Abdul-Nawfar, WS Wan-Azman, MS Arman-Z, S Abdul-Razak, MZ Rhendra-Hardy, WI Wan-Faisham

**Affiliations:** Department of Orthopaedics, Universiti Sains Malaysia, Kota Bharu, Malaysia; * Department of Plastic Surgery, Universiti Sains Malaysia, Kota Bharu, Malaysia; ** Department of Anaesthesiology, Universiti Sains Malaysia, Kota Bharu, Malaysia

**Keywords:** supracondylar humerus fracture, pink pulseless

## Abstract

The management of pink pulseless limbs in supracondylar fractures has remained controversial, especially with regards to the indication for exploration in a clinically well-perfused hand. We reviewed a series of seven patients who underwent surgical exploration of the brachial artery following supracondylar fracture. All patients had a non-palpable radial artery, which was confirmed by Doppler ultrasound. CT angiography revealed complete blockage of the artery with good collateral and distal run-off. Two patients were more complicated with peripheral nerve injuries, one median nerve and one ulnar nerve. Only one patient had persistent arterial constriction which required reverse saphenous graft. The brachial arteries were found to be compressed by fracture fragments, but were in continuity. The vessels were patent after the release of obstruction and the stabilization of the fracture. There was no transection of major nerves. The radial pulse was persistently present after 12 weeks, and the nerve activity returned to full function.

## Introduction

The incidence of brachial artery injury is not uncommon in supracondylar fractures. The risk has been reported at only 1%; however, recent studies noted an increase in vascular injury risks ranging from 12-15% ^1^,^2^. Vascular insufficiency that requires immediate surgical intervention is rare. The management of a pulseless well-perfused hand remains debatable and, to date, no consensus on management exists. Defensive strategy with closed observation had been advocated, however, some recommended early surgical exploration within the first 48 hours if pulselessness remains or compartment syndrome is evident. Reported complications of Volkmann contracture and loss of limb is low, yet it is still very important to clinically identify to prevent unnecessary and debilitating complications^3^.

## Materials and Methods

This is a retrospective study conducted from January 2011 to January 2016 on patients who were surgically treated for supracondylar humeral fracture with absence of radial pulse in a single institution. All seven patients had good hand circulation, but absent radial pulses were clinically evaluated and confirmed using Doppler signal. The brachial artery was explored in all patients, and fractures were finally stabilized with K-wires.

In this study, we reviewed the clinical presentation, injury pattern, CT angiography, and surgical findings at exploration of brachial artery. The final outcome was evaluated and relevant findings are presented.

## Results

**Fig. 1:**
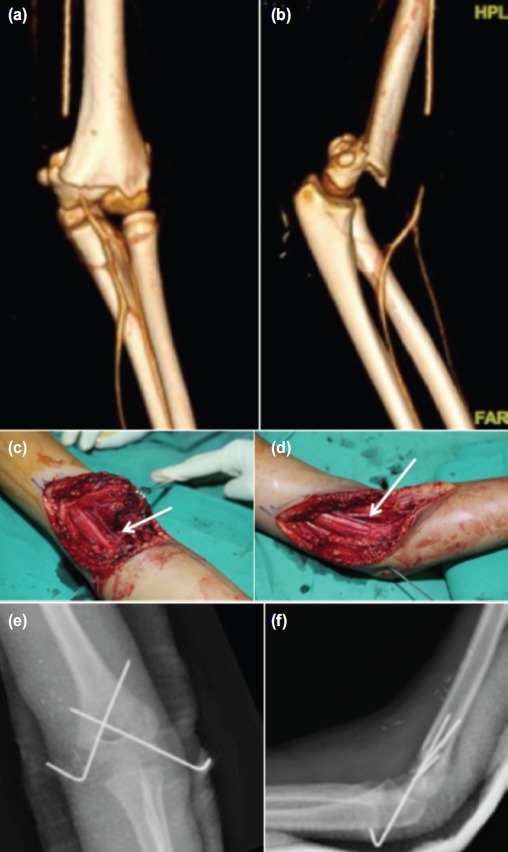
A 12 year-old boy with a supracondylar fracture. Pulses were not palpable and confirmed by Doppler; (a, b) CT angiogram revealed a complete block of the brachial artery with good collateral and distal run-off. (c, d) At operation, the brachial artery was found trapped and kinked by superior bony fragment. It was released and fracture was reduced. (e, f) The fracture was fixed with crossed K wires.

**Fig. 2:**
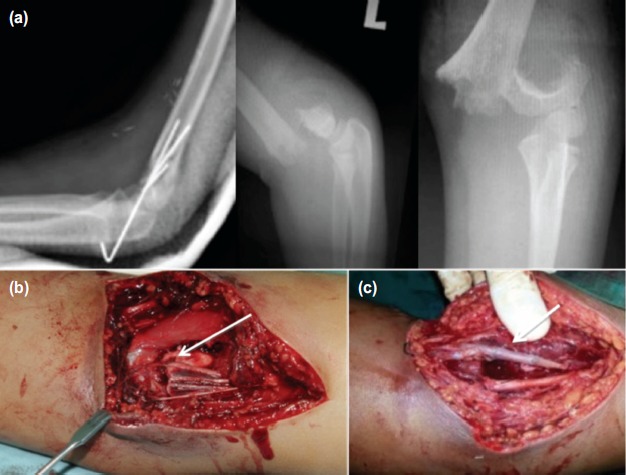
An 11 year-old boy with a displaced supracondylar fracture and absent distal pulses; (a) CT angiography revealed a 3cm segment loss of the brachial artery with good collateral. (b) The artery was trapped and constricted by the proximal fracture fragments. (c) Bypass with reverse saphenous graft was able to establish the flow.

**Fig. 3:**
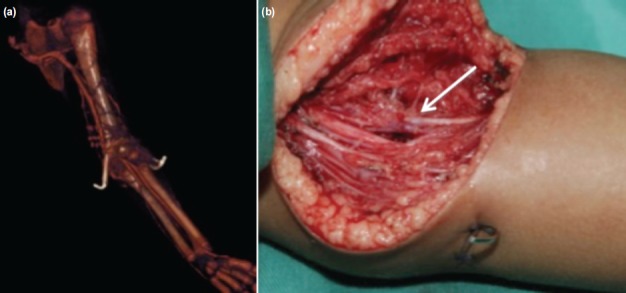
A 2-year old girl was referred with a pink and pulseless hand following a reduction of Gartland III supracondylar fracture; (a) CT angiogram revealed brachial artery block with good distal run-off. (b) Surgical exploration and tissue release were able to restore brachial artery flow and return pulses.

There were five males and two females with the mean age of 8.5 years-old (range 2-15 years). Extension type of fractures were noticed in all seven cases, and nerve injuries were associated in two. Radial pulse was not present in all patients, and an absent signal was confirmed on Doppler. The CT angiography revealed that four patients had a complete block but with the presence of collateral and distal run-off (Table I). All patients underwent exploration and release of brachial artery and K-wire stabilization of fracture through the anterior approach. The brachial artery was found compressed by proximal fragment fractures in all patients. The distal pulses returned to normal after the reduction and stabilization of the fracture, as well as the superficial adventitia release of contused segment arteries. Initial spasms of arteries were managed by the local application of verapamil or lignocaine (Fig. 1 - Case 7). The decision for reverse saphenous graft was made in one case for brachial artery constriction and contusion, in which the distal flow did not recover through local intervention (Fig. 2 - Case 6). The median time taken for the reestablishment of the pulse was 24 hours (range between 3-96 hours) with the mean absence of pulse to 31.4 hours. All supracondylar humeral fractures were stabilized by 1.6-1.8mm crossed K-wires. One patient had earlier undergone closed reduction and stabilization in another center, but the hand was subsequently pulseless. The CT angiograms revealed brachial artery block with good distal run-off. An immediate surgical exploration and release restored the arterial flow and distal pulses (Fig. 3 - Case 1). Median nerve injury and ulnar nerve palsy noted preoperatively were explored. Both nerves were contused and remained in continuity.

**Table I:** 

No.	Age	Sex	Type of Injury	Time to Reperfusion (Hour)	CT Angiography	Intraoperative Findings	Procedures	Bony	Neurodeficit	Neurological Deficit	Union	Other Complications
1	2	F	Extension on reduction and K-wire done	28	Yes-Block	Spasm	Exploration and release	K-wire	No	No	Complete	Nil
2	4	M	Extension	30	Yes-Block	Spasm	Exploration and release	K-wire	No	No	Complete	Nil
3	5	M	Extension	96	NA	Spasm	Exploration and release	K-wire	No	No	Complete	Nil
4	6	F	Extension	21	NA	Spasm	Exploration and release	K-wire	No	No	Complete	Nil
5	8	M	Extension	24	NA	Spasm	Exploration and release	K-wire	Neuropraxia ulnar nerve	Fully recovered at 5 weeks	Complete	Nil
6	11	M	Extension	18	Yes-Block	Constricted and spasm	Bypass	K-wire	No	No	Complete	Nil
7	12	M	Extension	3	Yes-Block	Spasm	Exploration and release	K-wire	Neuropraxia median nerve	Fully recovered at 6 weeks	Complete	Nil

M= Male, F= Female, NA= Not Available

Radial pulses were palpable, with pink and warm hands postoperatively in all seven patients and on discharge, and during follow-up. K-wires were retained for three weeks and fracture union was achieved after six weeks in all patients. No ischemic contracture was noted during the follow-up, and all patients with neurological deficit had fully recovered after at least six weeks post injury.

## Discussion

The management of pink pulseless hand in supracondylar fracture has long been controversial, especially in regard to the indication for exploration of the brachial artery in a clinically well-perfused full motor function hand. Griffith *et al,* in a literature review, concluded that the management depended largely upon clinical findings. A child with a pink pulseless hand following successful reduction could be managed conservatively. However, whenever additional signs of ischemia develop, surgical exploration was required^4^. A meta-analysis study by White *et al* concluded that absence of pulse after supracondylar humerus fracture is a strong indicator of vascular injury. Collateral circulation around the elbow can be variable and unreliable. Brachial artery revascularization in children can be successfully accomplished and has been associated with high success rate. Thus, the authors recommended an aggressive approach to absence of pulse by exploration and repair, as it is an indicator of vascular compromise, and the success rate for revascularization is sufficiently high. In contrast, Weller *et al *stated that in a pink pulseless hand, after closed reduction and stabilization, it is not an indication for vascular exploration provided the limb is well perfused. Close observation is mandatory to look for signs of deterioration^5^. A review questionnaire study of pediatrics orthopedic surgeons by Malviya *et al* revealed that only 16.1% of surgeons would proceed with exploration of vessels if pulses remain absent after stabilization^6^. The targeted vessels are often a few millimeters in diameter, and if they need surgical reconstruction, this requires microsurgical expertise. Furthermore, exploration carries the risk of injury to collateral vessels to maintain a viable extremity. Complications of symptomatic re-occlusion and residual stenosis are relatively high^7^. We explored all cases with loupe magnification and strict care was taken to not explore beyond levels of collaterals. The vessel reconstruction in an established thrombosed patient was performed under a microscope with a microsurgical expert. A recent paper by Louahem *et al* reviewed 68 pink pulseless hands of 404 Gartland III cases, and recommended urgent closed reduction of fracture followed by close monitoring. Circulation disturbances warranted immediate vascular exploration^8^.

Long-term late sequelae of ischemic forearm were reported by Blakey *et al* who suggested a more aggressive management to prevent major disability^3^. Recently, POSNA also recommended a more aggressive vascular intervention approach. Reviews further suggest a more aggressive vascular evaluation and exploration in selected cases^2^.

Luria *et al* reported 24 cases of supracondylar humerus fracture with vascular compromise. Out of 22 cases, immediate return of radial pulse was noted in 11 cases, and no return of pulses in another 11, after closed reduction and K-wiring. The patients without a return of pulse underwent primary exploration, and eight of them were found to have a tear or entrapment of the brachial artery^9^. Based on our findings, primary exploration without initial manipulation of six cases, only one case required vascular graft. We believe that improper attempt of closed reduction may further injure the vessels and lead to significant damage. Thus, it is safer to release the bony compression through exploration and open reduction. Our present study revealed that most cases can be managed by exploration and release of the vessels from the fracture site, thus returning the pulses and minimizing late complications of vascular injuries. The kinked vessels in children have shown to be more resistant to acute thrombosis, even though exploration was delayed with the mean duration of 24 hours. Only one patient required bypass graft due to acute thrombosis.

Nerve injury is also taken into consideration for immediate exploration of pulseless limbs. According to Luria *et al,* a significant correlation was found between median nerve deficit and vascular injury ^9^. Ramachandran suggested that coexisting ischemia is an indication for vascular exploration^10^. Mangat *et al* recommended early exploration of Gartland III supracondylar fracture in patients with coexisting anterior interosseous, or median nerve palsy, since these appear as a strong predictive indicator of nerve and vessel entrapment^11^. Both our cases showed complete recovery at about six weeks, indicating that the lesion was neuropraxia.

In conclusion, early exploration, open vascular release, and fracture reduction are options of treatment for pink pulseless hands in supracondylar fractures in children to maintain vessel patency.

## References

[1] Louahem DM, Nebunescu A, Canavese F, Dimeglio A. (2006). Neurovascular complications and severe displacement in supracondylar humerus fracture in children: Defensive or offensive strategy?. J Pediatric Orthop B.

[2] White L, Mehlman CT, Crawford AH. (2010). Perfused, pulseless, and puzzling: a systematic review of vascular injuries in pediatric supracondylar fracture humerus fractures and results of a POSNA questionnaire.. J Pediatric Orthop.

[3] Blakey CM, Bant LC, Birch R. (2009). Ischemia and the pink, pulseless hand complicating supracondylar fracture of the humerus in childhood- long-term follow-up.. J Bone Joint Sur Br.

[4] Griffin KJ, Walsh SR, Markar S, Tang TY, Boyle JR, Hayes PD. (2008). The pink pulseless hand. A review of the literature regarding management of vascular complication of supracondylar in children.. Eur J Endivasc Surg.

[5] Weller A, Garg S, Larson AN, Flethcer ND, Schiller JR, Kwon M. (2013). Management of pediatric pulseless supracondylar humerus fracture: Is vascular exploration necessary?. J Bone Joint Surg Am.

[6] Malviya A, Simmons D, Vallamshetia R, Bache CE. (2006). Pink pulseless hand following supracondylar fracture; An audit of British practice.. J Pediatric Orthop B.

[7] Sabharwal S, Tredwell SJ, Beauchamp RD. (1997). Management of pulseless pink hand in pediatric supracondylar of humerus.. J Pediatric Orthop.

[8] D Louahem (2016). J Cottalorda. Acute ischemia and pink pulseless hand in 68 of 404 Gartland type III supracondylar humeral fracture in children: Urgent management and therapeutic consensus. Injury.

[9] Luria S, Succar A, Eylon S. (2007). Vascular complication of supracondylar humeral fracture in children.. J Pediatric Orthop B.

[10] Ramachandran M, Birch R, Eastwood DM. (2006). Clinical outcome of nerve injuries associated with supracondylar fracture of the humerus in children: The experience of a specialist referral center.. J Bone Joint Surg Br.

[11] Mangat KS, Martin AG, Bache CE. (2009). The pulseless pink hand after supracondylar fracture of the humerus in children-The predictive of nerve palsy. J Bone Joint Surg Br.

